# Touchscreen-Based Cognitive Tasks Reveal Age-Related Impairment in a Primate Aging Model, the Grey Mouse Lemur (*Microcebus murinus*)

**DOI:** 10.1371/journal.pone.0109393

**Published:** 2014-10-09

**Authors:** Marine Joly, Sandra Ammersdörfer, Daniel Schmidtke, Elke Zimmermann

**Affiliations:** Institute of Zoology, University of Veterinary Medicine Hannover, Hannover, Germany; CNRS, France

## Abstract

Mouse lemurs are suggested to represent promising novel non-human primate models for aging research. However, standardized and cross-taxa cognitive testing methods are still lacking. Touchscreen-based testing procedures have proven high stimulus control and reliability in humans and rodents. The aim of this study was to adapt these procedures to mouse lemurs, thereby exploring the effect of age. We measured appetitive learning and cognitive flexibility of two age groups by applying pairwise visual discrimination (PD) and reversal learning (PDR) tasks. On average, mouse lemurs needed 24 days of training before starting with the PD task. Individual performances in PD and PDR tasks correlate significantly, suggesting that individual learning performance is unrelated to the respective task. Compared to the young, aged mouse lemurs showed impairments in both PD and PDR tasks. They needed significantly more trials to reach the task criteria. A much higher inter-individual variation in old than in young adults was revealed. Furthermore, in the PDR task, we found a significantly higher perseverance in aged compared to young adults, indicating an age-related deficit in cognitive flexibility. This study presents the first touchscreen-based data on the cognitive skills and age-related dysfunction in mouse lemurs and provides a unique basis to study mechanisms of inter-individual variation. It furthermore opens exciting perspectives for comparative approaches in aging, personality, and evolutionary research.

## Introduction

In humans there is strong evidence for inter-individual variability in the decline of cognitive ability with age. Normal cognitive aging has been well described and decline is not universal among the different cognitive domains but it is found in specific domains such as processing speed and reasoning, memory, and executive functions (for review see [1]). Sensitive and reliable cognitive testing procedures may reveal such impairments. Simple discrimination and reversal learning, for instance, have been used to investigate learning and cognitive flexibility and revealed age-related impairments in humans (e.g. [2], [3]), monkeys (e.g. [4]–[6]), and rats (e.g. [7]–[10]).

Promising novel non-human primate models for aging and age-associated diseases are mouse lemurs, the world's smallest non-human primates [11], [12]. Mouse lemurs are genetically more closely related to humans than rodents. They are nocturnal, solitary foragers while, during the day, they form sleeping groups [13], [14]. In the wild, mouse lemurs have a maximum lifespan of 8 years [15]. However, the life expectancy of mouse lemurs is higher in captivity: for our colony, the maximum recorded lifespan is 15 years. Due to their mouse-like body size, the maintenance and breeding of mouse lemurs is cost-efficient [11]. Therefore, they represent a valuable exception among primates for conducting long-term research, offering an ideal opportunity for studying their aging process not only cross-sectional, but also in longitudinal studies using individuals with known life history in existing aging colonies in captivity [11], [16]. Until now, studies on mouse lemurs have shown that cerebral atrophy is found in most aged animals [17], [18]. Brain pathologies similar to those of AD-patients can be found in some aged individuals [19], [20]; for review see [11], [12]. For example, Bons, Delacourte, and colleagues described β-amyloid plaques and pathological tau protein aggregation in *M. murinus* [19], [20]. Dhenain and colleagues later demonstrated iron accumulations in the mouse lemur brain as a process of non-pathological aging but with the same topography as in humans [21]. Besides biological and biochemical aspects of cerebral aging, studies on age-related cognitive decline in mouse lemurs are more limited. However, while no significant difference was observed between young adults and aged animals in cognitive tasks involving odour [22] or visual discrimination of light [23], some impairments were observed in more complex tasks such as shift and spatial rule-guided discrimination tasks [23]–[25]. A recent study demonstrated that mouse lemurs seem to be the only non-human primates reproducing the link between regional cerebral atrophy and age-associated cognitive alterations [23]. A decline in executive functions in aged mouse lemurs was associated with an atrophy of the septal region while impairment in spatial memory was correlated with atrophied hippocampo-entorhinal regions [23]. Most of the above-described studies used non-automated behavioural tasks, which impedes a direct comparison in translational research. Developing sensitive, reliable, and translational tasks to assess specific cognitive domains is, therefore, crucial to understand underlying neural mechanisms during aging.

The touchscreen testing method is a common procedure to assess cognitive abilities in humans and may help to easily detect age-related impairments in specific domains (e.g. the Cambridge Neuropsychological Test Automated Battery (CANTAB), a renowned battery of neuropsychological tests, see e.g. [26], [27]). The touchscreen testing method has several advantages such as high stimulus control, minimized operator-subject interaction, and a wide variety of cognitive tasks that can be performed using standardized testing procedures. A further advantage relevant for translational research is that touchscreen-based tests are adaptable to a broad range of animal species: animals may easily give responses to stimuli displayed on a screen by touching with the nose or hand. During the last decades, batteries of tests using a touchscreen testing method have been successfully used for assessing cognitive skills in rodents [28]–[32] as well as New World (marmoset: e.g. [33]) and Old World monkeys (rhesus monkey: e.g. [34], baboon: e.g. [35]). Some studies showed that age effects on executive functions can be reliably detected in both humans [26] and monkeys [36] using an automated method. To date, a computer-assisted translational approach that allows translating human-comparable cognitive tasks to mouse lemurs is missing. On the long run, such an approach would help to behaviorally and reliably test the functionality of specific brain regions of interest and to understand the mechanisms responsible for the great inter-individual variability in performance, i.e. discriminating healthy aging from neuropathological disorders.

In this study, we applied for the first time a touchscreen-based procedure to the model mouse lemur and examined different facets of cognition using two paradigms: a visual pairwise discrimination (PD) task and reversal learning (PDR). Learning the discrimination between two visual stimuli involves perceptual learning and non-hippocampal, associative stimulus-reward learning [37]. Since the reversal learning requires inhibition of the previously learned responses and the ability to learn the new stimulus-reward contingencies, the task assesses cognitive flexibility [37]. We hypothesized that, comparable to humans, monkeys, and rodents, young and aged mouse lemurs can be trained successfully to interact with a touch-sensitive screen and to respond for food reinforcement. Furthermore, we postulated that, comparable to humans, monkeys, and rodents, in computerized touchscreen-based tasks, age will have a significant effect on performance in the two cognitive tasks. We did not expect to find significant differences in attention and motivation levels during the tasks between the age groups.

Our study revealed that mouse lemurs can be trained successfully to use a touch-screen to get a reward within an average of 24 days. Individual performances in PD and PDR tasks correlate significantly, suggesting that individual performance is unrelated to the respective task. Compared to the young, aged mouse lemurs showed strong impairments in both PD and PDR tasks and high inter-individual variation. Attention and motivation did not differ between age groups in the respective tasks. Thus, our study provides the first touchscreen-based data on the cognitive skills and age-related dysfunction in the novel primate aging model mouse lemur. Findings open exciting perspectives for comparative approaches in aging, personality, and evolutionary research.

## Material and Methods

### Ethical statement

Experiments are non-invasive and belong to basic research. They may also be used to improve well-being under captive conditions. Experiments were performed in accordance with the NRC Guide for the Care and Use of Laboratory Animals, the European Directive 2010/63/EU on the protection of animals used for scientific purposes, and the German Animal Welfare Act. Our non-invasive testing procedure was approved by the Animal Welfare Committee of the University of Veterinary Medicine and approved and licensed by the Animal Welfare Committee of the Niedersächsisches Landesamt für Verbraucherschutz und Lebensmittelsicherheit (reference numbers: previously AZ 33.9-42502-05-10A080, now AZ 33.12-42502-04-14/1454, 28.04.2014).

All information mentioned is in accordance with the recommendations of the Weatherall report, “The use of non-human primates in research”.

We provided environmental enrichment to the mouse lemurs: within the housing cages, the animals of our colony have branches and hollow cylinders that allow them to climb and hide within their home cages. In addition, each cage is equipped with several sleeping boxes, to model the situation in nature, where mouse lemurs sleep, rest, and rear their offspring in tree holes [38].

### Subjects

**Figure 1:**
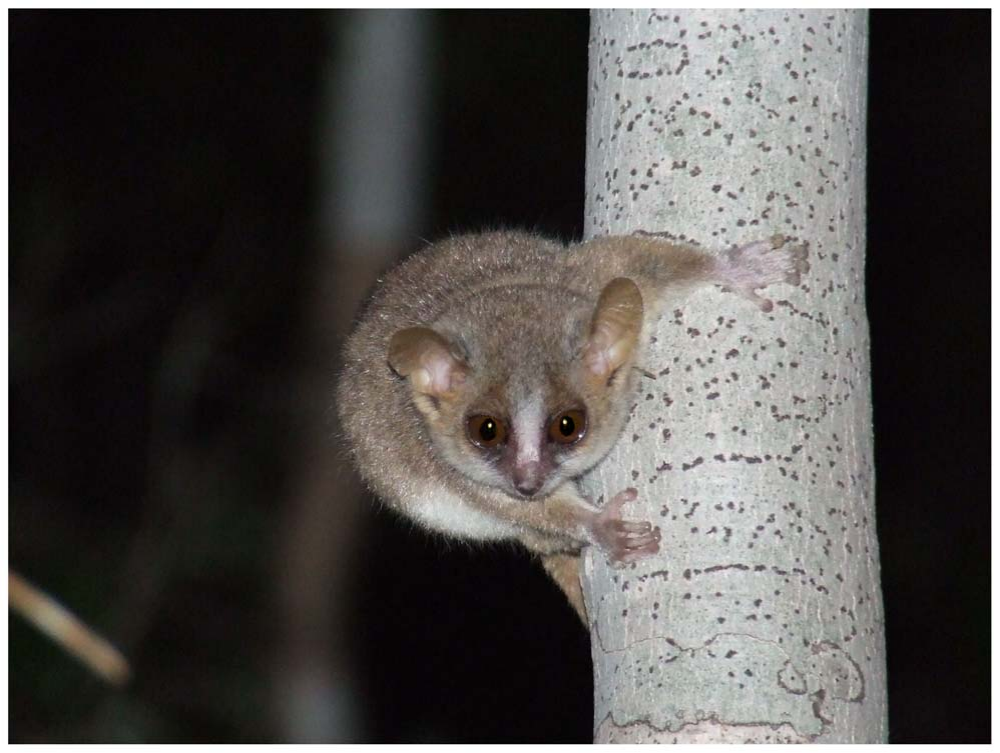
The grey mouse lemur (*Microcebus murinus*) represents the smallest primate aging model. Photograph taken by Dr. Christian Schopf.

Thirty adult grey mouse lemurs (*Microcebus murinus*, see Fig. 1), 14 males and 16 females, were included in this study (see Table 1). According to the age classification in previous studies [39], [40], we formed two age cohorts: one group of 20 young adults (10 males, 10 females; mean age  = 2.6 years, range: 1.1–4.1, see Table 1) and one group of 10 aged adults (4 males, 6 females; mean age  = 7.9 years, range: 6.9–9.5 years, see Table 1). Prior to the study, a veterinarian had checked the health status of each animal. An ophthalmologic examination was conducted, which allowed us to discard any individual with ocular pathology (approx. 1/3^rd^ of the aged animals from our colony; for methods see [41]).

**Table 1:** Performance of the mouse lemurs during the acquisition of the visual discrimination (PD) and the reversal learning (PDR).

				PD task					PDR task					
Subject	Sex	Age	Total number of training days	Positive image	Image bias in the first session	Number of sessions (trials) to reach the criterion	Response latency	Reward latency	Number of sessions (trials) to reach the criterion	Response latency	Reward latency	Number of perseverative errors (binomial criterion)	Number of perseverative errors (50% criterion)	Number of trials to reach 50% criterion
OTT	m	2.0	13	marble	-	5 (150)	5.156	1.619	11 (330)	4.255	1.448	64	110	180
PEA	m	2.6	48	fan	-	19 (570)	7.563	2.198	14 (420)	3.040	1.285	82	111	180
PED	m	2.1	24	fan	-	8 (240)	4.607	2.088	9 (270)	3.352	1.459	66	90	150
PEP	m	2.3	14	fan	-	7 (210)	2.481	0.919	15 (450)	2.071	0.972	50	157	270
PPP	m	2.0	31	marble	-	8 (233)	3.997	2.071	17 (426)	14.461	2.609	189	219	306
PHI	m	2.3	71	fan	marble	22 (614)*	12.296	27.673	25 (682)	17.415	24.229	65	159	263
PLU	m	2.1	25	marble	marble	3 (90)*	3.134	1.220	20 (570)	8.104	1.057	82	149	241
QUK	m	3.2	35	fan	marble	16 (470)*	11.559	4.475	16 (340)	19.330	59.989	78	150	250
QUN	m	3.0	11	marble	-	13 (381)	4.664	1.539	18 (540)	3.629	1.302	122	213	330
QUL	m	3.5	36	marble	-	19 (570)	3.635	1.410	26 (780)	2.012	0.865	100	164	270
NEL	f	1.2	38	marble	fan	12 (215)*	8.843	1.743	16 (381)	5.679	1.520	98	185	261
NOR	f	1.1	20	marble	-	4 (120)	5.040	1.286	13 (319)	17.903	2.559	40	127	229
PAM	f	3.1	26	fan	marble	18 (437)*	10.013	1.290	15 (358)	6.954	1.031	61	162	238
PAU	f	3.0	9	marble	-	12 (360)	3.785	1.802	32 (960)	3.676	1.015	216	367	600
PEG	f	2.5	17	fan	-	5 (150)	5.598	1.325	7 (210)	7.472	1.428	58	87	180
PEN	f	2.9	6	fan	-	6 (180)	5.433	2.976	9 (245)	20.064	2.830	67	108	154
PER	f	3.1	13	fan	-	9 (237)	9.946	1.787	7 (208)	3.705	1.749	46	84	148
POP	f	2.4	32	marble	-	4 (120)	3.117	1.405	11 (285)	43.976	32.011	27	96	165
QNI	f	4.1	10	marble	-	7 (210)	3.486	1.591	18 (540)	2.887	1.473	140	198	300
QEL	f	3.9	17	fan	-	6 (180)	7.460	0.935	13 (390)	7.641	0.982	102	191	330
ULI	m	7.8	12	fan	-	25 (574)	56.709	15.055	20 (600)	4.042	1.210	228	274	360
URB	m	7.5	20	marble	marble	6 (180)*	3.630	1.623	19 (539)	12.569	1.503	147	277	419
URI	m	7.4	33	marble	-	14 (420)	19.202	2.462	34 (1020)	8.803	1.539	89	434	750
VIR	m	8.6	22	fan	-	26 (780)	6.174	1.217	24 (617)	9.035	2.108	100	195	350
TIP	f	6.9	6	marble	-	19 (570)	2.844	3.348	33 (979)	6.530	1.398	447	493	769
UND	f	7.8	33	fan	-	8 (191)	10.928	2.888	14 (375)	5.896	2.531	14	152	284
URA	f	7.9	9	marble	-	10 (300)	5.037	1.796	11 (330)	8.666	1.408	73	122	180
URS	f	7.6	19	fan	-	9 (270)	5.379	1.314	9 (300)	4.420	1.714	64	91	150
VAN	f	8.4	14	fan	-	11 (330)	5.757	1.721	21 (630)	5.955	1.870	171	229	330
WIL	f	9.5	63	marble	-	26 (780)	3.061	2.678	60 (1800)	4.615	2.852	797	1001	1680

*Discarded from the performance analysis in the PD task because of an image bias.

All mouse lemurs used in this study were bred and kept in the colony of the Institute of Zoology, University of Veterinary Medicine Hannover [16], licensed for the maintenance and breeding of mouse lemurs (Erlaubnis gemäß §11 Abs. 1 Satz 1 Nr. 1 Tierschutzgesetz in Verbindung mit § 12 Tierschutz-Versuchstierverordnung, Landeshauptstadt Hannover, reference number AZ 42500/1H, 15.01.2014). The Zimmermann's mouse lemur colony was founded more than two decades ago at the University of Stuttgart-Hohenheim and it was the first colony in which the Goodman's mouse lemur (*Microcebus lehilahytsara*) has successfully been bred in captivity [16]. All subjects are registered in the mouse lemur studbook. The mouse lemurs used in the described study lived under a reversed, seasonally fluctuating light cycle (LD 14∶10 during the long-day period of 8 months; LD 10∶14 during the short-day period of 4 months) and were housed in different rooms where the dark phase, i.e. activity period, started either at 10:00 a.m., 12:00 a.m., or 2:00 p.m. In all rooms, the temperature and relative humidity were controlled and set to 23–25°C and 50–60%, respectively. Three times a week, the diet of the mouse lemurs consisted of seasonally changing fresh fruits and vegetables, dried fruits, nuts, as well as mealworms or locusts. Milk porridge enriched with vitamins, minerals, and albumin was offered the other 4 days of the week. During the study period, the tested mouse lemurs were maintained either alone or in pairs in cages of at least 0.75 m^3^ per animal. Animal weight was controlled every day and, on average, was 63±4 g during the testing period, with no significant differences between young and aged animals (Mann-Whitney U-test, N_young_  = 20, N_aged_  = 10, U = 85.0, p = 0.53). The weight at which mouse lemurs could be trained successfully corresponds to the weight under natural conditions (compare [16], [42], [43]). Adipose mouse lemurs had to be under a restricted diet to reach the normal weight before testing.

For this study, each mouse lemur was tested alone and on a daily basis ( = 1 session/day). Wild mouse lemurs are solitary foragers and separation from their sleeping partners during their activity phase corresponds to their natural behaviour [13], [14], [44]. Prior to the study, all animals were naïve to touch-sensitive screen devices.

### Test chamber

**Figure 2:**
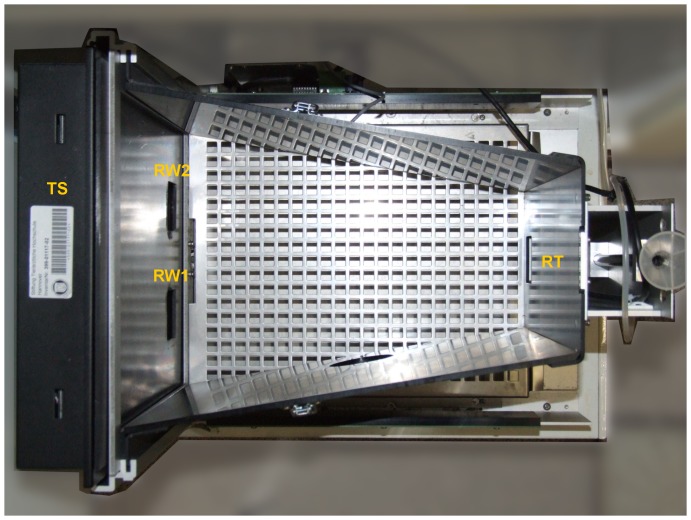
Test chamber (PD+ PDR task, top view). **TS** =  touchscreen device; **RW1**, **RW2** =  response windows; **RT** =  reward tray.

Experiments were performed in a separate testing room, containing a test chamber with a touch-sensitive screen (see Fig. 2). The test chamber was a customized version of the setup used by Bussey and colleagues [28] for rats (89540R-PD Task for rat Touch Screen System, Campden Instruments). The touch-sensitive screen could display visual stimuli in the front of the trapezoidal chamber (width: front  = 245 mm, back  = 130 mm; length  = 330 mm; height  = 95 mm), a reward tray delivering apple juice was in the rear wall of the chamber. The chamber was equipped with infrared photocells allowing the recording of entries in the reward tray. Each chamber output (touch on the screen and break of a photocell beam) was simultaneously recorded and analysed by a computer. A video camera and a video recorder were used to film and record each experiment for later offline analysis. A black Perspex mask with 2 response windows (46 mm×46 mm) allowed the mouse lemurs to give a response on one of 2 screen areas. Since mouse lemurs are nocturnal, both training and testing sessions were performed in the dark, i.e. all the visible lights that come with the basic version of the touchscreen setup (house and tray light) had permanently been deactivated.

### Training Protocol

The training and test protocols for rodents [28], [30], [31] were adapted to mouse lemurs. Mouse lemurs were trained and tested once a day ( = 1 session), 7 days a week, and within the first 2 hours of their nocturnal activity. The training stimuli were randomly drawn from a pool of 38 pictures (for a list see Fig. S1A). Each picture could only be drawn once per session. The training protocol was divided into the following 5 steps:

○ Step 1 - Habituation to the chamber: the animal was allowed to explore the chamber for 20 minutes. The reward tray was filled with 1 ml apple juice. This step lasted 1 session.
○ Step 2 – Initial training with images: two identical images were displayed on the screen for 30 seconds ( = one trial). The mouse lemur was rewarded as soon as it touched one image (75 µl apple juice), which made both images disappear, or after the two images disappeared automatically (25 µl apple juice). A reward was accompanied by the noise of the activated juice delivery pump. This noise was clearly perceived by the mouse lemurs (when first heard, all subjects showed ear movements and looked into the direction of the pump). A new trial started after a 10-second inter-trial interval (ITI). The maximal duration of a session was set to 30 minutes. We observed that mouse lemurs can adopt two different strategies in this step: either they actively interact with the screen within the 30 s and get a reward or they stay close to the reward tray to collect a reward after 30 s and wait there for the next trial/reward. We, therefore, defined two different criteria for this training step. For the first one, a given subject had to complete 30 trials in 20 minutes. This criterion could only be reached, when the subject interacted correctly with the touchscreen (1^st^ strategy). For those animals that adopted the second strategy, to simply collect the automatic reward without touchscreen interaction, the subject had to complete 30 trials in 30 minutes in 3 consecutive sessions. The interaction with the touchscreen then had to be learned in training step 3 (see below) by the latter animals.
○ Step 3 – Must touch one image: only 1 image was displayed at a pseudo-randomly chosen position (15× on the left side; 15× on the right side; never more than three consecutive trials on the same side), the other response window contained no image ( =  blank). The subject was rewarded with 25 µl apple juice as soon as it touched the image ( =  correct response). A new trial started when the mouse lemur collected the reward. A response to the blank screen was not rewarded ( =  incorrect response). A trial ended when the image was touched. Two consecutive trials were separated by a 10-s ITI. To reach the criterion for this step, the mouse lemur had to perform 30 trials in less than 30 minutes.
○ Step 4 – Must initiate a trial: as in step 3, only 1 image was shown at a pseudo-random position. To display the image (i.e. initiate the trial), the mouse lemur had to introduce its head into the empty reward tray. The mouse lemur was rewarded with 25 µl apple juice as soon as it gave a correct response. A trial ended when the image was touched. Two trials were separated by a 10-s ITI. To reach the criterion for this step, the mouse lemur had to perform 30 trials in less than 30 minutes.
○ Step 5 – Incorrect responses signalled by a tone: similar to step 4, but incorrect responses additionally were signalled by a 500-ms long 2000 Hz tone. In the case of incorrect responses, the trial was immediately stopped and the ITI was set to 15 s. To reach the criterion for this step, the mouse lemur had to complete 30 trials in 30 minutes with at least 80% correct responses on 2 consecutive days.

### Testing Protocol

Once the training was accomplished, mouse lemurs were tested in the Pairwise Discrimination (PD) task. The aim of this task is to evaluate the ability to discriminate two unknown images (white shapes on a black background). For all individuals of *M. murinus* trained in the pairwise discrimination and its reversal task, we used the “marble-fan” pair of visual stimuli that was also used in many studies investigating visual discrimination in mice ([30]; Fig. S1B). One image was arbitrarily used as the positive stimulus, the other one as the negative stimulus. The arbitrary choice was counterbalanced between individuals. The mouse lemur was rewarded with 25 µl apple juice as soon as it touched the positive stimulus. A touch on the negative stimulus was signalled by the same tone as in training step 5 and the increased ITI of 15 s. The ITI after correct choices was 10 s. A mouse lemur was considered discriminating both stimuli when it reached at least 80% correct responses in 2 consecutive sessions (maximum 30 trials per session). Within a session, both images were pseudo-randomly presented 15 times on the left and 15 times on the right side. The rewarded stimulus could not appear more than three consecutive trials on the same side. Once the mouse lemurs reached the PD task criterion (for a brief example video, see Movie S1), they were tested in the reversal task (PDR). The former positive stimulus was now the negative one and *vice-versa*. In the PDR, the criterion was identical to that in the PD task (i.e. at least 80% correct responses in 2 consecutive daily sessions).

### Data analysis

#### Training

The number of sessions required to complete each training step and the whole training phase were recorded for each mouse lemur. We then compared these numbers of sessions between young and aged adults using a Mann-Whitney U test. In general, we used the non-parametric Mann-Whitney U test for the comparisons made in this study, since our data has not uniformly been normally distributed (Shapiro-Wilk test).

#### PD task

Since visual stimulus bias may affect the data analysis and interpretation in PD [30], we assessed for each mouse lemur, whether it showed a preference or an aversion for the positive stimulus in the first session using a binomial test. Six mouse lemurs (3 young males, 2 young females and 1 aged male) showed such a stimulus bias in the first session (see Table 1). Most of them showed a positive bias for the “marble” image (3 young males, 1 young female and 1 aged male), 1 for the “fan” image (1 young female). The performance data for these animals were discarded from the performance analysis in the PD task.

The performance of a mouse lemur was assessed using a learning curve with the percentage accuracy for each session and by counting the number of sessions and trials as well as errors an animal needed to reach the task criterion. The performance of both age groups was compared using a Mann-Whitney U test. Within the group of young animals, we also tested for an influence of sex (male *vs.* female) using a Mann-Whitney U test.

Lastly, we controlled for differences in attention and motivation during the task between both age groups [30]. For that purpose, the average latency to respond after the stimuli display and the average latency to collect the reward after giving a correct answer was calculated for each mouse lemur and over all sessions. We compared these measurements between young and aged adults using a Mann-Whitney U test. Within the young subjects, we again tested for an influence of sex (male *vs.* female) using a Mann-Whitney U test.

#### PDR task

In the first session, using a binomial test, we checked whether mouse lemurs showed a bias for the former PD positive stimulus, as it would be expected. As for the PD task, the performance of each mouse lemur was then assessed using a learning curve with the percentage accuracy for each session and the number of sessions and trials an animal needed to reach the task criterion. Furthermore, we analysed the perseveration phase as defined by Mar and colleagues [31]. For this purpose, using a binomial test, we determined for each session, whether a mouse lemur persevered and responded significantly more to the formerly positive stimulus. The perseverative phase, therefore, included all sessions in which the mouse lemurs showed a response bias for the formerly positive stimulus. Within the perseverative phase, the number of errors, so-called perseverative errors, was counted for each animal. As a second measure for the cognitive flexibility/perseverance of the subjects during reversal learning, we counted the individual number of trials needed and errors made until reaching a criterion of 50% correct trials (chance level) in two complete, consecutive sessions. This criterion was taken to define the definite end of perseverance and the starting point of the new learning phase.

The performance and the number of perseverative errors of both age groups were compared using a Mann-Whitney U test. Within the young animals, we also tested for sex differences (male *vs.* female) using a Mann-Whitney U test.

Lastly, we controlled for differences in attention and motivation during the task between both age groups [31]. For that purpose, the average latency to respond after the stimuli display and the average latency to collect the reward after giving a correct answer over all sessions were calculated for each mouse lemur. We compared these measurements between young and aged adults using a Mann-Whitney U test. Within the group of the young subjects, we also tested for an influence of sex (male *vs.* female) using a Mann-Whitney U test.

When not indicated, results are given in Mean ±SEM. The level of statistical significance was set at p = 0.05. A trend was considered when 0.05<p≤0.1. All statistical tests were exact and calculated using STATISTICA 10 (StatSoft, Hamburg, Germany).

## Results

### Training

All mouse lemurs were successfully trained to interact with the touch-sensitive screen. They mostly used their snout but also both snout and one hand or only one hand to touch the screen. They needed an average of 24.2±2.9 days of training before entering the PD task (see Table 1). Training step 2 was completed in 4.1±0.7 days, step 3 in 6.0±1.4, step 4 in 2.9±0.6, and step 5 in 10.2±1.5 days. No significant differences in the total number of training days, nor in the number of days needed to complete each different training step was found between young and aged mouse lemurs (Mann-Whitney U test, U≥87, N_young_  = 20, N_aged_  = 10, p≥0.588). Great inter-individual variability in the number of days required for training was found in both age groups (young range: 6–71; aged range: 6–63; see Table 1).

### PD task

**Figure 3:**
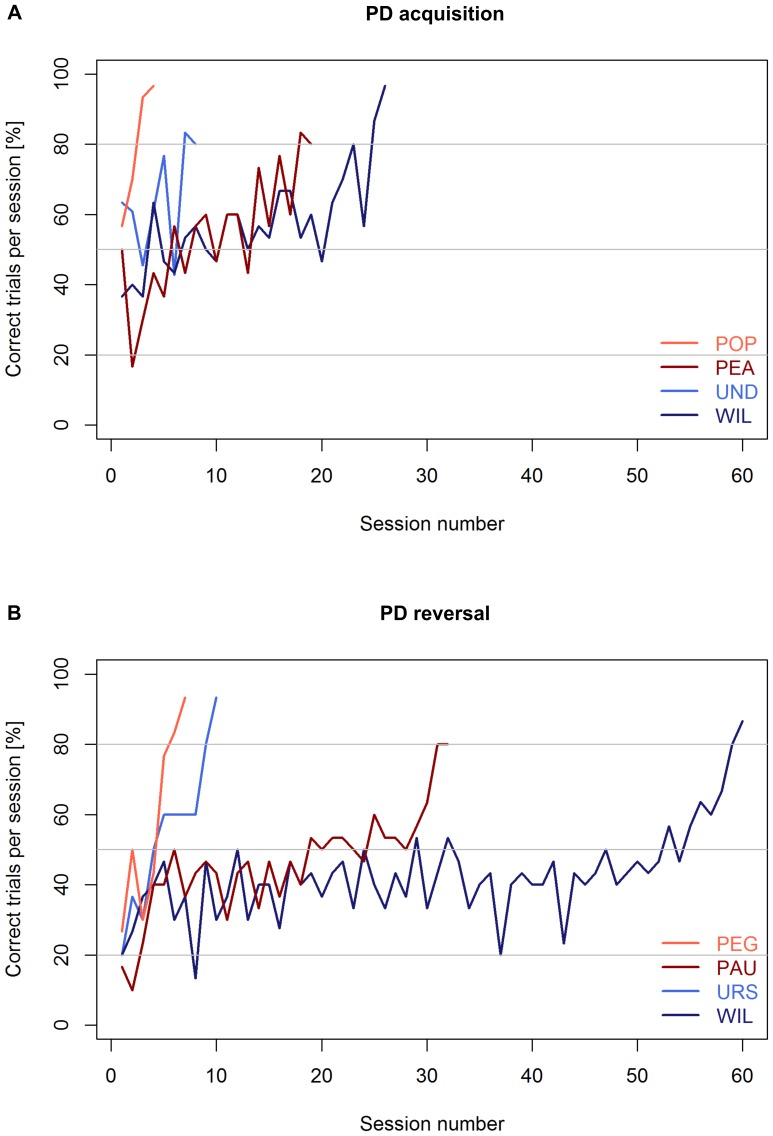
Representative learning curves of young and old mouse lemurs in the visual pair-wise discrimination task (PD) and its reversal (PDR). **A** Learning curves of a good (POP, UND) and a bad (PEA, WIL) **PD** learner from each age category (red  =  young subjects; blue  =  old subjects). **B** Learning curves of a good (PEG, URS) and a bad (PAU, WIL) **PDR** learner from each age category (red  =  young subjects; blue  =  old subjects).

**Figure 4:**
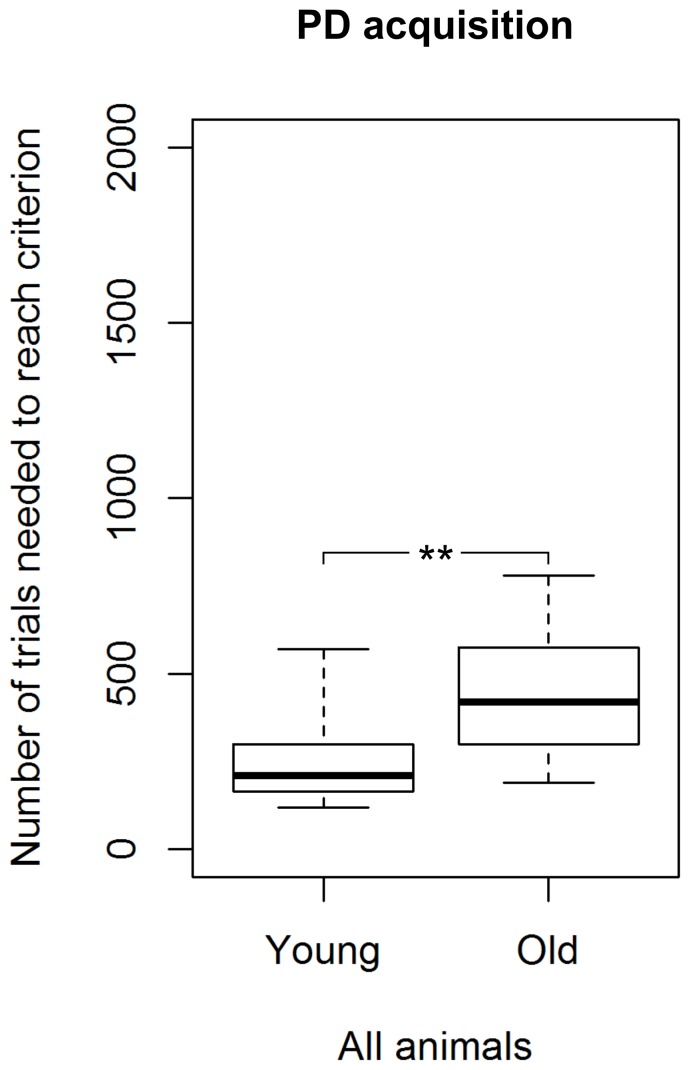
Performance of young and aged mouse lemurs in the visual discrimination task (PD). ** Indicates a significant difference with p<0.01. N_young_  = 15, N_aged_  = 9. The box represents the lower quartile, median, and upper quartile, the whiskers represent the minimum and maximum values.

**Figure 5:**
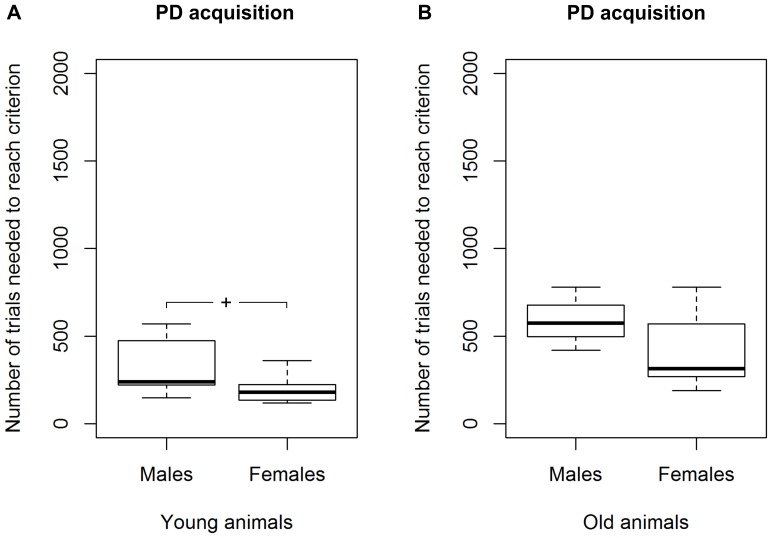
Performance of males and females in the visual discrimination task (PD). **A** Results for the young mouse lemurs and **B** results for the aged mouse lemurs. **^+^** Indicates a trend with 0.05<p<0.1. N_young males_  = 7, N_young females_  = 8, N_aged males_  = 3, N_aged females_  = 6. The box represents the lower quartile, median, and upper quartile, the whiskers represent the minimum and maximum values.

All tested mouse lemurs succeeded in acquiring the visual discrimination (see Table 1; for examples of learning curves see Fig. 3A). Among the 24 mouse lemurs that showed no spontaneous preference for an image, we found a significant difference in the number of sessions and trials required to reach the criterion between young and aged adults (Mann-Whitney U test, N_young_  = 15, N_aged_  = 9, U≤24, p≤0.009). Young mouse lemurs needed fewer trials (median  = 210) than aged ones (median  = 420) to reach the criterion (Fig. 4). Great inter-individual variability in the number of trials to reach the criterion was found: the performance of young adults ranged from 120 to 570, while it was even wider (range: 191–780) for the aged ones (Fig. 4). Among young adults, we found that females tended to require fewer trials to reach the criterion than males (Mann-Whitney U test, N_males_  = 7, N_females_  = 8, U = 11, p = 0.054; Fig. 5A). Due to the smaller sample size, aged adults could not reliably be statistically compared for sex differences in the median number of trials needed to reach the PD-criterion. Nevertheless, aged females also showed better values than aged males (Fig. 5B).

### Control for attention to the task

Young and aged mouse lemurs did not differ significantly in their latency to respond after the stimuli display (median_young_  = 4.7 s, range: 2.5–9.9 s; median_aged_  = 5.8 s, range: 2.8–56.7 s; Mann-Whitney U test, N_young_  = 15, N_aged_  = 9, U = 46, p = 0.215). Among young adults, we found no significant differences in the latency to respond between the sexes (Mann-Whitney test, N_males_  = 7, N_females_  = 8, U = 22, p = 0.536). Among aged adults, females showed a shorter median latency to respond than males (median_females_  = 5.2 s, range: 2.8–10.9 s; median_males_  = 19.2 s, range: 6.2–56.7 s). However, the sample of the aged males was too small to statistically verify this effect.

### Control for motivation to collect the reward

Young mouse lemurs tended to collect the reward quicker than the aged ones (median_young_  = 1.6 s, range: 0.9–3.0 s; median_aged_  = 2.5 s, range: 1.2–15.1 s; Mann-Whitney test, N_young_  = 15, N_aged_  = 9, U = 39, p = 0.096), but this statistical trend was mainly caused by the contribution of one of the old males (ULI).

### PDR task

**Figure 6:**
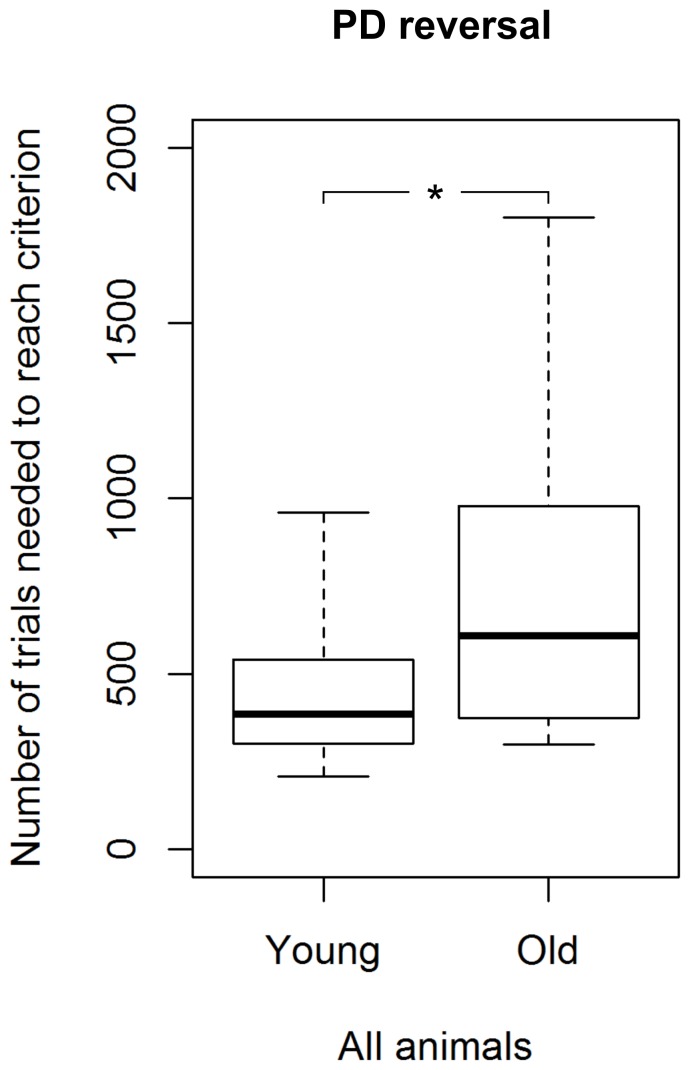
Performance of young and aged mouse lemurs in the reversal learning task (PDR). * Indicates a significant difference with p<0.05. N_young_  = 20, N_aged_  = 10. The box represents the lower quartile, median, and upper quartile, the whiskers represent the minimum and maximum values.

**Figure 7:**
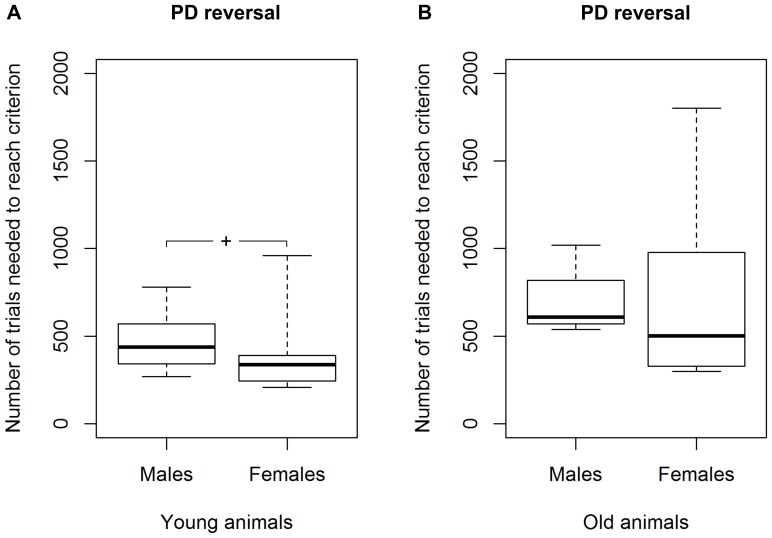
Performance of males and females in the reversal learning task (PDR). **A** Results for the young mouse lemurs and **B** results for the aged mouse lemurs. **^+^** Indicates a trend with 0.05<p<0.1. N_young males_  = 10, N_young females_  = 10, N_aged males_  = 4, N_aged females_  = 6. The box represents the lower quartile, median, and upper quartile, the whiskers represent the minimum and maximum values.

**Figure 8:**
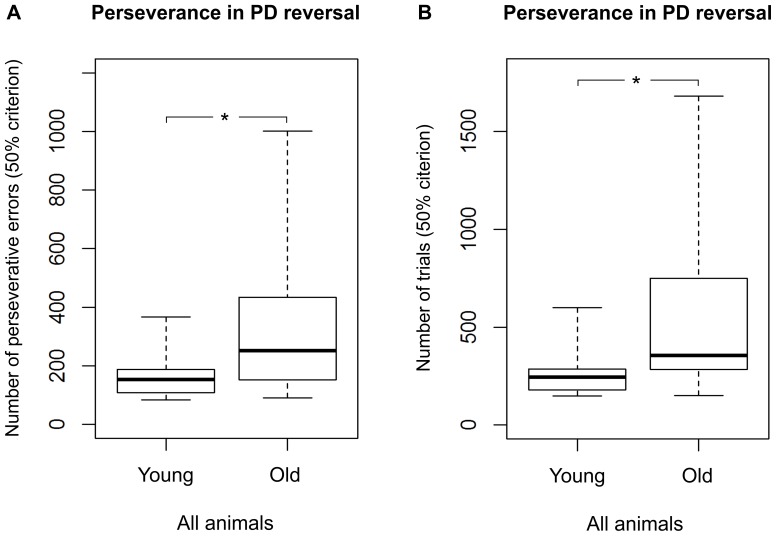
Perseverance of young and aged mouse lemurs in the reversal learning task (PDR). **A** Number of trials needed to reach the 50% criterion in the PDR. **B** Number of perseverative errors made until reaching the 50% criterion in the PDR. * Indicates a significant difference with p<0.05. N_young_  = 20, N_aged_  = 10. The box represents the lower quartile, median, and upper quartile, the whiskers represent the minimum and maximum values.

All 30 tested mouse lemurs succeeded in the reversal learning (see Table 1; for examples of learning curves see Fig. 3B). In the first session, the average accuracy was 20.3±1.8%. We found a statistical trend for a difference in the number of sessions (p = 0.061) and a significant difference in the number of trials required to reach the criterion between young and aged adults (Mann-Whitney U test, N_young_  = 20, N_aged_  = 10, U = 54.5, p = 0.044; Fig. 6). Young mouse lemurs needed fewer trials (median  = 390) than aged ones (median  = 617) to reach the criterion (Fig. 6). Again, great inter-individual variability in the number of trials to reach the criterion was found: the performance of young adults ranged from 208 to 960, while it was much wider (range: 300–1800) for the aged ones. Among the young adults, we found that females tended to require fewer trials (median  = 302) to reach the criterion than males (median  = 426; Mann-Whitney U test, N_males_  =  N_females_  = 10, U = 27.5, p = 0.089; Fig. 7A; for the results of the aged animals, see Fig. 7B). Aged mouse lemurs tended to make more perseverative errors (binomial criterion) than young ones (median_young_  = 72, range: 27–216; median_aged_  = 123, range: 14–797; Mann-Whitney U test, N_young_  = 20, N_aged_  = 10, U = 60, p = 0.082). Using the 50% criterion to measure perseverance revealed that aged and young adults differed significantly in both the total number of perseverative errors (median_young_  = 153.5, range: 84–367; median_aged_  = 251.5, range: 91–1001; Mann-Whitney U test, N_young_  = 20, N_aged_  = 10, U = 47, p = 0.019; Fig. 8A) and the number of trials (median_young_  = 245.5, range: 148–600; median_aged_  = 355, range: 150–1680; Mann-Whitney U test, N_young_  = 20, N_aged_  = 10, U = 43, p = 0.011; Fig. 8B). On the group level, aged adults needed more trials to quit following the former rule and, accordingly, made more wrong decisions before ultimately re-reaching chance performance.

Within the group of the young animals, no significant sex differences in the number of perseverative errors were found for any of the criteria (Mann-Whitney U test, N_males_  =  N_females_  = 10, U≥40, p≥0.481).

### Correlation between PD and PDR performance

**Figure 9:**
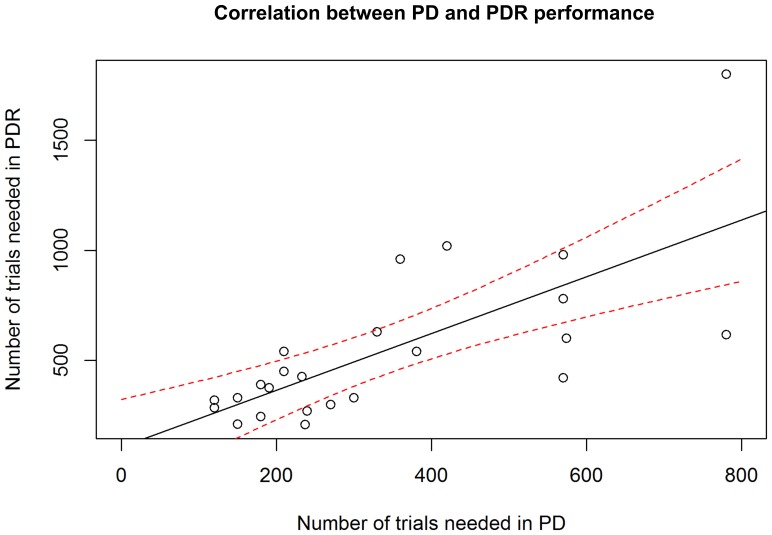
Correlation between individual PD and PDR performances. Number of trials needed to reach criterion in the reversal learning task (PDR) plotted against the number of trials needed to reach criterion in the visual discrimination task (PD) (N_total_  = 24). The solid black line represents the regression line, the dashed red lines represent the 95% confidence interval as estimated from the linear regression model. The individual performances in PD and PDR are highly significantly correlated (Spearman's rank correlation, r = 0.72, p = 0.00007).

We found a highly significant, positive correlation (Spearman's rank correlation, r = 0.72, p = 0.00007) between the individual performances in the PD and PDR tasks, i.e. subjects that needed a high number of trials to reach the criterion in the visual discrimination task also needed a high number of trials to reach the criterion in the reversal task (Fig. 9)

### Control for attention to the task

Young and aged mouse lemurs did not differ significantly in their latency to respond after the stimuli were displayed (median_young_  = 6.3 s, range: 2.0–44.0 s; median_aged_  = 6.2 s, range: 4.0–12.6 s; Mann-Whitney U test, N_young_  = 20, N_aged_  = 10, U = 88, p = 0.619). We did not find any significant sex differences in this variable within the young animals (Mann-Whitney U test, N_males_  =  N_females_  = 10, U  =  35, p  =  0.280).

### Control for motivation to collect the reward

Young and aged mouse lemurs did not significantly differ in their latency to collect the reward (median_young_  =  1.5 s, range: 0.9–60.0 s; median_aged_  =  1.6 s, range: 1.2–2.9 s; Mann-Whitney U test, N_young_  =  20, N_aged_  =  10, U  =  81, p  =  0.422). No significant sex differences in the median reward latency were found within the young animals (Mann-Whitney U test, N_males_  =  N_females_  =  10, U = 44, p = 0.684).

## Discussion

Our findings revealed for the first time that young and aged mouse lemurs could be successfully trained on a visual pair-wise discrimination task and its reversal using a touchscreen standardized automated system. We found age-associated cognitive decline in the acquisition of the visual discrimination as well as in the reversal learning. We, thus, demonstrated for the first time the successful use of a standardized touchscreen method and cross-species comparative approach to assess age-related cognitive impairments in mouse lemurs.

Interestingly, we found that aged mouse lemurs, as a group, were impaired in the acquisition of a visual discrimination between two images. This result supports comparable findings in humans [3] and monkeys [36] but contrasts with previous findings on mouse lemurs in a visual discrimination task [23]. While the procedures used in the human and monkey studies were comparable to our mouse lemur study (i.e. touchscreen-based testing method using discrimination between images [3], [36]), the visual discrimination task of Picq and colleagues [23] differed in two details: firstly, in the study of Picq and co-workers, mouse lemurs were placed in a work chamber where they had to choose between two corridors leading to a reinforcement chamber. The visual discrimination had to be made on the basis of the illumination of the corridors (S+  =  illuminated corridor; S-  =  dark corridor). In the visual discrimination task used for our study, mouse lemurs were required to (1) learn to perceptually discriminate two white shapes on a black background and to (2) learn which of the two shapes was associated with the reward. Thus, differences in the difficulty of the visual discrimination may have led to the diverging results. Secondly, the main motivation for the subjects in the study of Picq and colleagues was to reach the safety of a nest box. By choosing a food reward for our study, the conditioning paradigm in our case was clearly appetitive and differences in the conditioning procedure might also have contributed to the differing observations.

The fact that all our mouse lemurs succeeded in the visual discrimination task, shows that they were able to perceptually discriminate both images. Thus, using a standardized and automated testing procedure, our results revealed for the first time that aged mouse lemurs show cognitive impairments that have so far only been found in aged humans and monkeys. Since all mouse lemurs were checked for eye diseases, corneal consistence, integrity of retina, and intraocular pressure of each eye prior to the study, we can exclude differences in the perceptual abilities between the age groups in our study [41]. We found a strong inter-individual variability in the learning performance. The oldest animal (WIL, 9.5 years old) had the worst learning performance while other aged mouse lemurs (e.g. VAN, 8.4 years old) performed just as good as young ones. This task based on a touchscreen testing method has been proven to be sensitive to dysfunctions in the perirhinal cortex of the medial temporal lobe of rats [45]. The temporal lobe, including the secondary visual area, is actually one of the regions that are the most affected by age-dependent atrophies in mouse lemurs [23]. A magnetic resonance imaging study is underway to explore whether a link between brain morphology and cognition can be confirmed in our subjects.

We found that aged mouse lemurs, as a group, were also impaired when faced with the reversed reward contingency (PDR task). Reversal learning procedures are widely used to assess flexibility or behavioral adjustment to changing rules [31]. In this task, mouse lemurs not only had to learn to extinguish the previously rewarded response but also to choose the previously unrewarded image. We found significant differences in perseverance measures in the PDR between the two age cohorts, indicating that aged subjects persevere more in their errors, i.e. they had difficulties to ignore the previously rewarded response and to flexibly adapt to the reversed stimulus-reward contingency. This result is in line with previous findings in aged mouse lemurs [23], [24], aged rats [10], aged monkeys [6], and aged humans [3] and, thus, may reflect a common pattern in brain aging across mammals. The PDR task has proven to be sensitive to the integrity and functionality of the orbitofrontal cortex in rats and monkeys [9], [46]. Orbitofrontal lesions in rats and monkeys and lesions of the dorsolateral striatum in mice significantly slow down visual reversal learning [9], [46], [47]. Lesions of the medial prefrontal cortex in rodents may also impair the reversal learning, but only when visual stimuli are difficult to discriminate [47]. Again, our current magnetic resonance imaging study in mouse lemurs will help to illuminate potential links between atrophies in these brain regions and cognition in the same subjects.

Within young mouse lemurs, we reported that females seemed to perform better than males in acquisition of the visual discrimination and reversal learning. Although the sample size was small, aged females also tended to be better than males of comparable age (see Fig. 5B). A larger sample will help to confirm sex differences in cognitive functions and to explore the potential influence of sex hormones on learning abilities (see for instance the effect of hormonal status on spatial memory in female chimpanzees [48]) in future studies.

Lastly, we provide future prospects for the study of visual acuity in nocturnal small-brained primates using the same behavioural tasks as in rodents, non-human, and human primates. We showed that mouse lemurs could solve a visual discrimination of shapes. Through the visual discrimination of white shapes on a black background, we gained behavioural insight into the visual acuity of mouse lemurs, since they were clearly able to make their choice from the rear end of the chamber. Mouse lemurs, thus, can visually discriminate white shapes of less than 4 cm^2^ from a distance of more than 20 cm. To our best knowledge, there is only one study that has previously reported data on visual acuity in the grey mouse lemurs: Dkhissi-Benyahya and colleagues estimated visual acuity anatomically [49], using peak retinal ganglion cell density and spacing, and found an acuity value of 4.2 cycles/degree, which is higher than for rats (about 1 cycle/degree see [50]). It confirms that visual acuity of nocturnal mammals, usually considered as poor, actually shows a great variability between species. The computer-assisted standardized method described here may also be used to conduct further behavioural investigations on the visual acuity of mouse lemurs that might provide valuable information concerning the evolution of the visual system in nocturnal mammals.

To conclude, we successfully adapted a touchscreen based testing method for the assessment of age-related impairments in non-hippocampal, associative learning and cognitive flexibility (a component of executive functions) to the use in the grey mouse lemur, *M. murinus*, a novel primate model for aging. Based on that, the development and adaptation of further cross-taxa touchscreen-based automated attention, learning, and memory tasks for mouse lemurs will help to assess further facets of cognition and its disorders and to embed the learning and memory capacity of these early primates into the evolution of primate intelligence.

## Supporting Information

Figure S1 **List of visual stimuli used.** **A** 38 different stimuli have been used during the training procedure. **B** Pair of stimuli (“marbles”  =  left; “fan”  =  right) used for the actual visual discrimination task and its reversal.
(TIF)

Movie S1 **Example video of a young subject in the visual discrimination task (PD)**. The video starts in the middle of a session and shows how the animal completes 4 trials of the 30 trial session. The touchscreen and the two response windows can be seen at the upper end of the field of view (FOV), whereas the reward tray is located outside the FOV at the opposite side of the chamber.
(WMV)
