# Supplementary figures and images for: Touchscreen-Based Cognitive Tasks Reveal Age-Related Impairment in a Primate Aging Model, the Grey Mouse Lemur (Microcebus murinus)
Source: PLoS One. 2014 Oct 9;9(10):e109393. doi: 10.1371/journal.pone.0109393 (PMC4192115; doi:10.1371/journal.pone.0109393)

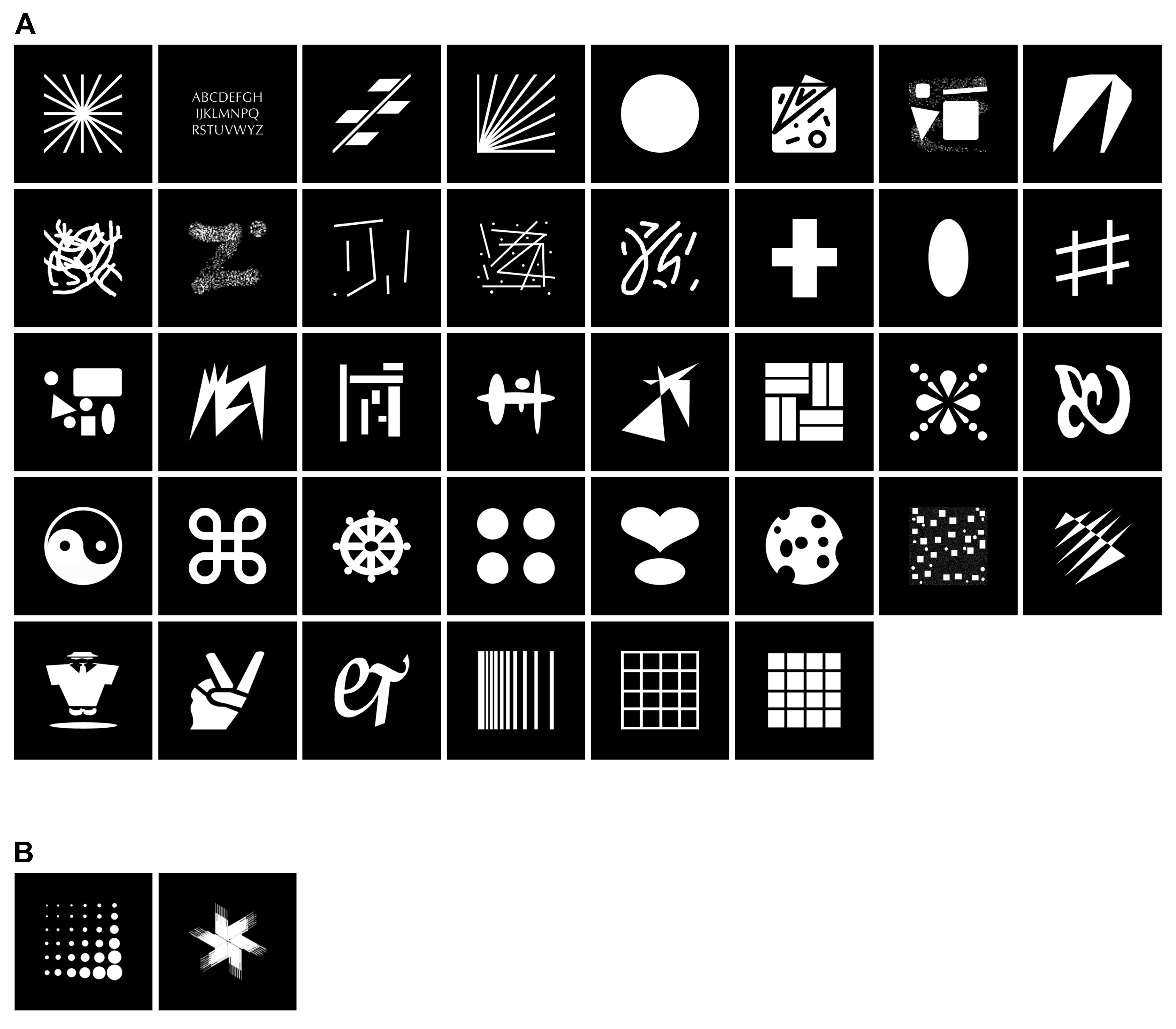

Supplement: Figure S1 — List of visual stimuli used. A 38 different stimuli have been used during the training procedure. B Pair of stimuli (“marbles” = left; “fan” = right) used for the actual visual discrimination task and its reversal. (TIF) [file pone.0109393.s001.tif]
